# Global Hemostatic Methods to Tailor Treatment With Bypassing Agents in Hemophilia A With Inhibitors— A Single-Center, Pilot Study

**DOI:** 10.1177/10760296241260053

**Published:** 2024-07-25

**Authors:** Roza Chaireti, Nida Soutari, Margareta Holmström, Pia Petrini, Maria Magnusson, Susanna Ranta, Iva Pruner, Jovan P. Antovic

**Affiliations:** 1 Department of Molecular Medicine and Surgery, 27106 Karolinska Institutet, Stockholm, Sweden; 2 Coagulation Unit, Department of Haematology, Karolinska University Hospital, Stockholm, Sweden; 3 Department of Medicine, Solna, 27106 Karolinska Institutet, Stockholm, Sweden; 4 Clinical Chemistry, Medical Diagnostics, Karolinska University Hospital, Stockholm, Sweden; 5 Department of Health, Medicine and Caring Sciences, 4566 Linköping University, Linköping, Sweden; 6 Pediatric Coagulation Unit, Astrid Lindgren Children's Hospital, Karolinska University Hospital, Stockholm, Sweden; 7 Division of Pediatrics, Department of Clinical Science Intervention and Technology, 27106 Karolinska Institutet, Stockholm, Sweden; 8 Department of Women's and Children's Health, 27106 Karolinska Institutet, Stockholm, Sweden

**Keywords:** hemophilia A, activated prothrombin complex concentrate, recombinant factor VIIa, thrombin generation, inhibitor

## Abstract

For patients with hemophilia A and high-titer inhibitors treated with bypassing agents there are no reliable methods to assess treatment effect. We investigated the utility of global hemostatic methods in assessing treatment with bypassing agents (rFVIIa or activated prothrombin complex [aPCC]). All patients with hemophilia A and inhibitors followed at the Coagulation Unit or the Pediatric Coagulation Unit at Karolinska University Hospital aged 6 years and above were eligible for this noninterventional study. Baseline plasma samples were spiked with bypassing agents in increasing concentrations (aPCC 50 U/kg, 100 U/kg, 150 U/kg, and rFVIIa 90 μg/kg and 270 μg/kg) in vitro. For patients treated with factor concentrates or bypassing agents follow-up samples were collected (in vivo tests). The samples were analyzed using overall hemostatic potential (OHP), and calibrated automated thrombogram, Calibrated Automated Thrombogram (CAT). Nine patients with hemophilia A with inhibitors were included. Spiking with rFVIIa normalized the coagulation potential in 6/8 samples, in 3 only with high dose. Only one sample did not improve adequately after spiking with aPCC. The improvement in hemostasis was reliably shown by both CAT and OHP. The baseline potential was, however, more often measurable by OHP compared to CAT. Factor concentrate had been administered to 5 patients normalizing the hemostatic potential in vivo in 2 (without spiking). The hemostatic improvement induced by spiking with rFVIIa or aPCC is shown by OHP and CAT, but the results have to be evaluated in larger cohorts.

## Introduction

Hemophilia is a congenital, X-linked bleeding disorder characterized by deficiency of coagulation factor VIII (FVIII, hemophilia A [HA]) or IX (FIX, hemophilia B), which results in impaired thrombin generation (TG).^1^ The cornerstone of treatment is replacement of the missing coagulation factor, either prophylactically or on-demand.^2^ Early introduction of treatment with factor concentrates in recent decades has contributed to decreased morbidity and mortality, as well as improved quality of life, especially in severe hemophilia.^3^ Development of neutralizing antibodies (inhibitors) against the coagulation factor concentrates impair effectiveness of the replacement therapy necessitating different treatment approach such as bypassing agents (activated prothrombin complex [aPCC] and activated factor VII [rFVIIa]),^4,5^ οr more recently in HA, emicizumab.^6^

The management of patients with hemophilia with inhibitors depends on the severity of bleeding symptoms, titer, and the type of inhibitor^7^ and often requires personalized treatment regimen. The effects of treatment with bypassing agents, expressed by the clinical phenotype, have shown considerable variations among patients.^8^ However, personalization of treatment requires robust follow-up and evaluation of the effect and there is, to date, no reliable routine laboratory method. Global hemostatic methods, used mainly in research, have so far shown promising results as assessment tools for treatment efficacy and follow-up. Methods such as TG assays (TGA)^9^ and thromboelastography^10^ have been used in different patient cohorts with varying results.^11^ Although the introduction of emicizumab has led to promising results in retaining a good hemostatic response,^12^ bypassing agents are still recommended as treatment of acute bleedings or as preoperative supplement.^13^ Also issues such as cost and availability can affect prophylactic treatment decisions. Different study designs, cohort sizes, and treatment protocols have made standardization of follow-up and subsequent treatment tailoring difficult.

The present, single-center study investigates the effect of different concentrations of bypassing agents on the hemostatic potential of adult and pediatric patients with congenital HA and inhibitors in vitro and in vivo. The aim of the study is to assess global hemostatic methods as a tool to personalize treatment with bypassing agents in order to supplement the evaluation of the clinical effect.

## Patients and Methods

### Patients

Patients with congenital HA and inhibitors aged ≥6 years and followed at the Coagulation Unit, Department of Haematology, and the Pediatric Coagulation Unit, Karolinska University Hospital were eligible for inclusion. All patients received oral and written information and signed the informed consent form prior to inclusion. For patients younger than 18 years of age, informed consent was also given by the patient's legal guardian(s).

The detailed study protocol is presented at https://clinicaltrials.gov (*NCT02453542*)*.* In short, blood samples were drawn in connection with routine sampling, both prior and following administration of bypassing agents, or FVIII concentrates. Bloods samples were taken from each patient at multiple occasions.

The following data were collected from the patients’ digital medical records: demographics (age, gender), data on hemophilia and bleedings (type, inhibitor levels, target joint), and treatment at the time of the sampling and response to treatment.

In this manuscript, we used international nonproprietary names (INNs) instead of commercial brand names for all medications but Immunate, which has no INN since it is a plasma-derived FVIII concentrate.

### Blood Sampling and Spiking

Blood samples were collected by venipuncture and collected in tubes containing 0.13 mM citrate (Vacutainer, Becton Dickinson) and immediately centrifuged at 2000 *g* for 15 min. After collecting the supernatant, plasma was recentrifuged for another 15 min at 2000 *g*. Platelet-poor plasma (PPP) was stored at −70 °C until analyzed.

Plasma samples were spiked with aPCC and/or rFVIIa at increasing concentrations (for aPCC 50 U/kg, 100 U/kg, and 150 U/kg and for rFVIIa 90 μg/kg and 270 μg/kg). In cases where the available plasma volume was low, only 2 concentrations were chosen for aPCC (50 U/kg, 100 U/kg).

We calculated the concentrations for aPCC according to the experiment conducted by Livnat et al^14^ where a final concentration of 0.8 U/mL, 1.2 U/mL, and 1.6 U/mL in the sample corresponds to a dosage of 50 U/kg, 75 U/kg, and 100 U/kg, respectively. The concentrations for rFVIIa were calculated according to the experiments presented by Antovic and Antovic^15^ where 2.4 μg/mL and 9.6 μg/mL correspond to a dosage of 90 μg/kg, respectively, 270 μg/kg.

If the patient received treatment at the outpatient clinic (Coagulation Unit) with factor concentrate or bypassing agent and subsequent sampling were performed after c:a 30 min. Those samples were analyzed without spiking and the results are referred as “in vivo tests.”

### Routine Analyses

FVIII and inhibitor levels were measured by the routine methods used at the Coagulation Laboratory, Clinical Chemistry, Karolinska University Hospital, that is, a chromogenic method and the Bethesda method,^16^ respectively.

### Calibrated Automated Thrombogram

The Calibrated Automated Thrombogram (CAT) method has been previously described.^17^ In short, the frozen PPP was thawed by immersion into a water bath at 37 °C immediately prior to analysis. The same procedure was followed for all samples. Thrombin generation was measured by the CAT method as described in the Thrombogram Guide by Thrombinoscope BV. The mixture of PPP reagent LOW (trigger) and PPP used in the assay contained 1 pM tissue factor and 4 μM phospholipids. The markers calculated were lagtime (clotting time, the moment at which TG begins, in minutes), endogenous thrombin potential (ETP, the total amount of generated thrombin, in nanomolar*minute, nM*min), peak height (maximal thrombin concentration, Cmax, in nanomolar thrombin, nM), time to peak height (ttpeak; ie, time to reach maximal thrombin concentration, in minutes). All reagents were obtained from Thrombinoscope BV, Maastricht, The Netherlands. The 96-well plates used were obtained from Ninolab, Stockholm, Sweden.

Five samples were analyzed by reducing both reagents and plasma samples by 50% according to the description by,^18^ namely “midiCAT.” This was done for practical reasons, since the large volume of plasma required for the global hemostatic methods and the repeated spikings would otherwise limit the analyses which could be performed.

The reference range for CAT in our laboratory is: lagtime 3.33-11.83 min, ETP 980.5-2837.4 nM*min, peak 94.78-415.54 nM, and ttpeak 5.67-17 min (24 women and 16 men, average age 30.5 years).

We considered an increase in ETP to the levels stated in the study on mild hemophiliacs by Trossaërt et al^19^ to be satisfactory, that is, 1060 ± 450 nM*min.

### Overall Hemostatic Potential

The overall hemostatic potential (OHP) was measured as described previously.^20^ In short, repeated spectrophotometric measurements of the fibrin aggregation curve (ie, absorbance) in PPP were recorded in the presence of small amounts of exogenous thrombin, tissue-type plasminogen activator, and calcium chloride. The fibrin aggregation curve reflects both the gradual conversion of the fibrinogen into fibrin by thrombin and the production of plasmin by the activation of plasminogen, which in turn digests fibrin. Each absorbance (Abs) value is indicative of the level of fibrin at a particular time point, and the area under the curve (AUC) reflects the balance between fibrin generation and lysis period. The AUC is calculated as the sum of Abs readings. The OCP (overall coagulation potential) represents the AUC for the fibrin generation curves, whereas the OHP is calculated as the sum of AUC for both the fibrin generation and lysis curves as described in Vranic et al.^21^ The overall fibrinolysis potential (OFP) represents the difference between the OHP and OCP curves and is presented as percentage of OCP. The reference range is: OHP 6.5-13.6, OCP 12.7-24.0, and overall fibrinolysis potential (OFP%) 31% - 57%.^22^

### Statistical Analysis

We used descriptive statistics to present the results. Due to few complete measurements for CAT and considering that the study is based on real-world samples, that is, as taken in the clinical practice without any intervention, we opted for not performing statistics for CAT. We used nonparametric tests (Mann-Whitney *U* test) to compare the results between the spiking experiments with aPCC and rFVIIa for OHP. A *P*-value <.05 was considered significant. All analyses were performed by the IBM SPSS Statistics v.25.

## Results

### Patients

Nine patients were included, 2 of whom were adults at the time of inclusion. The median age at inclusion was 10 years (range 6-79). All patients had a severe bleeding phenotype, with >3 bleedings/year and 7 had target joints. Some of the patients were treated with immune tolerance induction at time of blood sampling (see Table 1). The type and dosage of hemostatic treatment varied greatly. The median titer of inhibitors at inclusion (first sampling) was 12 (range 1.2-3000) Bethesda Units (BU), at the second sampling 456 (1.3-2910) BU, and at the third sampling 323 (1-1755) BU. Multiple blood samples were included for 5 patients (Table 1). Results from global hemostatic methods performed on a total of 18 blood samples were included in the study (Table 1).

**Table 1.** Patient Characteristics.^a^

Patient number	Age at Sampling (Years)	ITI at Sampling	Antibody Titer at Sampling (BU)	Treatment at Sampling	Time (hours) Between Latest Treatment and Sampling	Plasma Level of FVIII at Sampling	Plasma Level of FVIII Following Treatment* (in vivo)	Treatment for in vivo Testing
First blood sampling								
1	12	Yes	14	Immunate 3000 U (62 U/kg) once daily	15	0.008 kIU/L	NA	NA
2	58	No	19	aPCC 3000 U (36 U/kg) once daily	48	NA	NA	NA
3	79	No	5.8	aPCC 2000-4000 U (32-64 U/kg) on demand	NA	NA	NA	NA
4	10	No	25	rFVIIa 5 mg on demand (178 μg/kg)	12	NA	NA	NA
5	11	No	1.2	Immunate 2000 U (51 U/kg) once daily	12	NA	NA	Immunate 2000 U
6	8	No	12	NA	NA	<0.005 kIU/L	<0.005 kIU/L	Turoctocog alfa 3000 U
7	8	No	2	Wilate 4000 U (138 U/kg) once daily	24	<0.005 kIU/L	1.37 kIU/L	Wilate 4000 U
8	8	Yes	3000	Simoctocog alfa 2000 U (100 U/kg) twice daily	12	NA	NA	Simoctocog alfa 2000 U
9	6	No	8.9	rFVIIa 2 mg (125 μg/kg) on demand	NA	<0.005 kIU/L	<0.005 kIU/L	rFVIIa 2 mg
Second blood sampling								
1	13	Yes	1.3	Immunate 4000 U (83 U/kg) once daily	12	0.007 kIU/L	0.96 kIU/L	Immunate 4000 U
2	58	No	NA*	aPCC 3000 U (34 U/kg) 3 times daily	12	NA	NA	NA
4	12	Yes	688	Simoctocog alfa 6000 U (187 U/kg) once daily	24	<0.005 kIU/L	NA	rFVIIa 5 mg (156 μg/kg)
6	8	Yes	224	Turoctocog alfa 3000 U (260 U/kg) on demand	NA	<0.005 kIU/L	<0.005 kIU/L	Turoctocog alfa 3000 U
8	8	Yes	2910	Simoctocog alfa 2000 U (100 U/kg) twice daily	12	<0.005 kIU/L	<0.005 kIU/L	Simoctocog alfa 2000 U
Third blood sampling								
1	14	Yes	1	Immunate 4000 U (81 U/kg) once daily	12	<0005 kIU/L	0.97 kIU/L	Immunate 4000 U
4	12	Yes	610	Simoctocog alfa 6000 U (175 U/kg) once daily	24	<0.005 kIU/L	NA	Simoctocog alfa 6000 U
6	8	Yes	36	NA	NA	<0.005 kIU/L	<0.005 kIU/L	Turoctocog alfa 3000 U (125 U/kg)
8	6	Yes	1755	Simoctocog alfa 2000 U (90 U/kg) twice daily	12	<0.005 kIU/L	0.007 kIU/L	Simoctocog alfa 2000 U

Abbreviations: aPCC, activated prothrombin complex; ITI, immune tolerance induction.

a The time between sampling 2 and 3 for patient nr 2 was 2 days and the antibody titer was not measured.

### Calibrated Automated Thrombogram for Spiked Samples

The baseline hemostatic potential, as expressed by TG, was unmeasurably low in 14 of 18 samples, limiting the number of complete analyses and making statistical analyses irrelevant. The increases mentioned in Table 2 are based on the 4 analyses with valid baseline values. CAT markers (refer to Methods, CAT) following spiking were available for additionally 5 analyses.

**Table 2.** Median (Range) Increase in OCP and OHP, as Well as Decrease in OFP Between Baseline and After Spiking With aPCC (50 U/kg, 100 U/kg, 150 U/kg) and rFVIIa (90 μg/kg, 270 μg/kg) in Patients With Congenital Hemophilia A With Inhibitors.

	aPCC 50 U/kg	aPCC 100 U/kg	aPCC 150 U/kg	rFVIIa 90 μg/kg	rFVIIa 270 μg/kg
OCP	22.1 (2.9-2320)	24.3 (3.5-1183)	22 (2.9-1694.8)	17.5 (2.5-942.1)	22.2 (3-970.5)
OHP	4.2 (4-221.7)	4.6 (4.1-209.6)	2 (0-198)	2 (0-249)	2 (0-241.6)
OFP%	0.02 (0.01-15.3)	0.008 (0.006-12.2)	0	0.007 (0-16.3)	0

Abbreviations: aPCC, activated prothrombin complex; OCP, overall coagulation potential; OHP, overall hemostatic potential.

The increase of ETP was satisfactory (>1060 ± 450 nM*min) for 8 of 9 analyses at a spiking dosage corresponding to 50 U/kg aPCC and 3 of 8 (one missing) at a spiking dose corresponding to 90 μg/kg rFVIIa. For the sample with low ETP at a dosage 50 U/kg aPCC, spiking concentration of 100 U/kg and 150 U/kg aPCC did not increase ETP to satisfactory levels. For 3 of the 5 analyses where ETP was low at rFVIIa 90 μg/kg, administration of high dosage of rFVIIa 240 μg/kg resulted in satisfactory ETP. Refer to Table 3 for values.

**Table 3.** Median (Range) Values of AUC (ETP), Cmax, Lagtime, and Ttpeak (Time to Cmax) at Baseline and After Spiking With aPCC (50 U/kg, 100 U/kg, 150 U/kg) and rFVIIa (90 μg/kg, 270 μg/kg) in Patients With Congenital Hemophilia A With Inhibitors.

	AUC (nM*min)	Cmax (nM)	Lagtime (min)	Ttpeak (min)
Baseline	340.92 (78.57-573.94)	16.1 (3.64-33.69)	7.5 (5-8.17)	19 (15.83-25.83)
aPCC 50U/kg	794.39 (198.74-1502.78)	47.49 (13.48-85.7)	3.83 (2.83-6.33)	13.83 (11.5-19.67)
aPCC 100 U/kg	986.38 (413.14-1755.14)	47.13 (29.66-107.65)	4.17 (3,17-6.5)	14.67 (10.83-17.5)
aPCC 150 U/kg	1561.32 (76.71-2066.38)	89.82 (3,41-134.29)	3.83 (3.67-5)	13.67 (11.83-18)
rFVIIa 90 μg/kg	615.66 (278.47-1642.75)	27.94 (16.86-114.52)	4.59 (3.17-6.17)	16.92 (13.67-18.83)
rFVIIa 270 μg/kg	796.15 (459.26-2311.09)	41.95 (24.37-125.74)	5 (3.83-6.83)	17.17 (15.33-20.67)

Abbreviations: aPCC, activated prothrombin complex; AUC, area under the curve; ETP, endogenous thrombin potential.

### Overall Hemostatic Potential for Spiked Samples

The changes in the hemostatic potential were calculated for 16 of 18 samples (2 missing samples where the baseline hemostatic potential was unmeasurably low). Overall hemostatic potential normalized for 16 of 18 analyses already at a spiking dosage corresponding to 50 U/kg aPCC and 15 of 15 (3 missing) at a spiking dose corresponding to 90 μg/kg rFVIIa. The median increases in OHP and OCP and decreases in OFP are shown in Table 2.

No statistically significant differences were noted between the increases in OHP and OCP and decreases in OFP for the different concentrations of aPCC and rFVIIa, or between aPCC and rFVIIa.

### Overall Hemostatic Potential for in Vivo Tests

Of the 18 samplings which were spiked, valid results for concomitantly taken in vivo samples were available for 4 patients with 5 samplings: (1) patient 1, sample 2 (in vivo with Immunate® 4000 U), (2) patient 2, sample 2 (in vivo with aPCC 3000 U), (3) patient 5, sample 1 (in vivo with Immunate 2000 U), and (4) patient 6, samples 1 and 2 (in vivo with turoctocog alfa 3000 U in both cases) (Table 4).

**Table 4.** FVIII Levels, Treatment, and OHP and CAT Parameters for the in Vivo Results.^a^

Pat	Age (Years)	FVIII 1 (kIU/L)	FVIII 2 (kIU/L)	Antibody Titre (BU)	Treatment	OCP 1	OCP 2	OHP 1	OHP 2	OFP 1 (%)	OFP 2 (%)
1	13	0.008	NA	1.3	Immunate 4000 U	0.1	11.6	0.06	9.9	58.7	14.9
2	56	NA	NA	NA	aPCC 3000 U	1.3	6.6	0.3	6.1	78.6	8.1
5	11	NA	NA	1.2	Immunate 2000 U	1.4	23.2	1.9	13.8	0	40.5
6.1	8	0.005	0.005	12	Turoctocog alfa 3000 U	0.02	0.05	0	0	255.3	96.3
6.2	8	0.005	0.005	224	Turoctocog alfa 3000 U	0.2	0.1	0	0	103.4	117.4

Abbreviations: aPCC, activated prothrombin complex; CAT, Calibrated Automated Thrombogram; OCP, overall coagulation potential; OHP, overall hemostatic potential.

a 1: Before and 2: After Treatment.

In patients 1 and 5, OHP was normalized (from baseline 0.06 to 9.9 and from baseline 1.85 to 13.8, respectively) following administration of Immunate, similarly to spiked samples. In patient 2, OHP increased, but was not completely normalized following administration of 36 U/kg aPCC in vivo (0.3→6.1), whereas it was normalized in vitro following spiking with corresponding dosage (50 U/kg). In patient 6, there was no increase in OHP following administration of turoctocog alfa, whereas OHP was normalized following spiking with both bypassing agents at the lowest dosage.

## Discussion

The challenges of treating a patient with hemophilia and inhibitors are accentuated by the difficulties to measure the effect of administered bypassing agents. Our results support the previous literature by showing that TGA and OHP can be used to test in vitro response of bypassing agents in baseline samples to supplement the clinical decisions. We chose to use plasma samples taken without using a standard wash-out period to focus on the actual increase of the hemostatic potential in real-world patients, especially given that, in some cases, the extremely low hemostatic potential made it impossible for the methods to produce a measurement.

It has previously been showed that both TGA and OHP correlate with factors levels and phenotype in patients with all severities of HA, both with and without inhibitors.^23–26^ In our study, levels of FVIII at baseline where very low and even the increases, when available after administration of factor concentrates, were most often marginal. However, there are data supporting that the results can correlate with the phenotype, which would be the next step in our study. The effect of rFVIIa added to plasma from patients with HA and HB with and without inhibitors can be detected by OHP up for to 2 h following administration, corresponding to some intensive treatment protocols.^23,27^ Repeated measurements of spiked plasma can contribute to further information on the duration, as well as the extent of hemostatic improvement.

The ability of TGA to respond to changes in management has been shown previously, regardless of the intervention.^9,28^ Additionally, total and peak thrombin concentration can differentiate between moderate and severe hemophilia and is thus clinically relevant.^24^

We chose to focus on the total TG (ETP) since previous studies have shown that ETP and peak (“concentration markers”) are the most relevant biologically. Additionally, it corresponds better to disease severity and phenotype in contrast to the “time marker” ttpeak which correlates with factor levels in patients with hemophilia.^27,29,30^

However, decrease and in many cases normalization of both lagtime and ttpeak were observed in our study, indicating a consistent improvement in hemostasis.

Dose tailoring is not a new concept. Other groups have previously spiked blood samples obtained from patients with hemophilia with inhibitors to examine which dosage led to a normalized hemostatic potential and used the results to treat those patients.^31,32^ Other studies have shown improvements in hemostasis when using different combinations of bypassing agents and concentrates.^33,34^ In our study, we did not have enough valid results from the blood samples taken for in vivo testing, and we cannot yet establish an association with certainty. However, among the few samples tested we observed an improvement in the hemostatic potential when spiking with bypassing agents in vitro which was not observed in the after-treatment samples where factor concentrates were administered, especially if the patient had high antibody titers (>5 BU). Spiking with bypassing agents could be a part of the initial assessment of patients with hemophilia with inhibitors. In that case, the clinician will be aware of which bypassing agent works best in-vitro for the individual patient and use it in cases of acute bleedings. In our study, we observed that, although the hemostatic potential was improved by both bypassing agents used in most cases, there were differences in the dosage required, and the grade of improvement, that is, one bypassing agent could be deemed potentially better than the other in an individual patient. Whether this corresponds to the clinical picture remains to be seen, but we have already used these results in our clinical practice with promising results. For example, the differences in the in vitro response led us to switch the bypassing agent administered to patient nr 8, which improved both the bleeding phenotype and the severe hemarthroses that this patient suffered from. After the change in prophylactic treatment from rFVIIa to aPCC he could, after 3 months, walk with a crutch instead of being wheelchair bound (results presented as oral communication at the 51^s^ Nordic Coagulation meeting in Stockholm, September 6-8, 2018).

One of the weaknesses of the study is the small size of the cohort. We plan to expand the study by including more centers, thereby increasing credibility and applicability of the results. However, considering the rarity of hemophilia and the lack of robust standardized data, we nonetheless consider our results to be a valuable contribution to the field. Another weakness is the fact that the patients had different baseline hemostatic potentials, since we did not define a wash-out period as prerequisite for blood sampling and received different treatments. This meant that it was not optimal to study the actual coagulation status prior and after administration of the bypassing treatment for the patients as a group. We tackled this by studying improvement, rather than levels of coagulation markers. Additionally, there were some missing values, including cases when the baseline (pretreatment) coagulation potential was so low that it could not be recorded. Assay times longer than what is currently used (ie, 60 min) and potentially longer incubation time with the bypassing agent during spiking might improve the results. The fact that a wash-out period was not required prior to blood sampling and that blood samples were acquired at the time of the routine sampling means that all data and results from our cohort was real-world data and reflect the actual clinical situation and challenges.

In conclusion, our study contributes with additional data on the usage of global hemostatic methods to tailor treatment. We suggest that spiking of samples with different concentrations of bypassing agents can assist the clinician in making decisions on changing treatment if there are signs of treatment failure. Blood samples can be obtained and analyzed at any time, without a standard wash-out period. Longer assay times might improve the calculations, especially at baseline.
